# Distinguishing post-COVID from long-COVID in adults: Development and validation of a biomarker signature using targeted proteomics and machine learning in a cross-sectional observational study

**DOI:** 10.1371/journal.pone.0338451

**Published:** 2026-02-27

**Authors:** Franziska Meyer, Stephan Traidl, Milad Ameri, Anita Dreher, Nevine Abu-Rashed-Kufs, Jan Vontobel, Matthias Möhrenschlager, Hans-Werner Duchna, Felicia Sandberg, Marie-Charlotte Brüggen

**Affiliations:** 1 Hochgebirgsklinik Davos, Davos, Switzerland; 2 Faculty of Medicine, University of Zurich, Zurich, Switzerland; 3 Allergy Unit, Department of Dermatology, University Hospital Zurich, Zurich, Switzerland; 4 Department of Dermatology and Allergy, Hannover Medical School, Hannover, Germany; 5 Christine Kühne-Center for Allergy Research and Education, Davos, Switzerland; 6 Swiss Institute for Allergy and Asthma Research, Davos, Switzerland; 7 Cardio-CARE, Medizincampus Davos, Davos, Switzerland; Independent Medical Researcher and Writer, UNITED KINGDOM OF GREAT BRITAIN AND NORTHERN IRELAND

## Abstract

**Background:**

COVID-19 can have diverse clinical manifestations, ranging from asymptomatic infection to critical illness with multiorgan involvement. While many patients recover fully, others develop long-COVID, a heterogeneous condition marked by persistent symptoms beyond the acute phase. The immunological pathomechanisms between long-COVID and other post-acute recovery states remain unclear.

**Objective:**

To characterize and compare clinical, pulmonary, and proteomic profiles of patients with long-COVID (LC) and those recovering from severe COVID-19 without long-COVID (post-severe-COVID, PC), and to evaluate the predictive potential of machine learning–based biomarker analysis.

**Methods:**

In this monocentric, prospective observational study with a cross-sectional design, patients undergoing rehabilitation were included at admission. Clinical data, detailed symptom profiles, and lung function testing, including diffusing capacity of the lungs, were collected. Serum proteomics covering immune response and inflammation panels was performed, and a Random Forest classifier was applied to identify biomarkers differentiating LC and PC.

**Results:**

LC (n = 24) patients were younger (52 years vs. 58 years in PC), predominantly female (66.7% vs. 30.0% in PC), and reported fatigue, neurocognitive symptoms, and exercise intolerance, whereas PC (n = 40) patients showed greater pulmonary impairment, as shown by reduced diffusing capacity (46% vs. 72.5% in LC *p*<0.001). Proteomic profiling revealed distinct immune and inflammatory signatures between groups. Applying a random forest classification algorithm, we were able to distinguish between the LC and the PC group with a high degree of accuracy of around 89%, using LAMP3 (Lysosome-associated membrane glycoprotein 3), CKAP4 (cytoskeleton associated protein 4) and KRT19 (Keratin 19).

**Conclusions:**

This study introduces a novel characterization of patients recovering from severe COVID-19 without long-COVID, enabling clearer differentiation between persistent and recovering trajectories. Combining clinical data, pulmonary function, and proteomic machine learning analysis provides insight into post-acute COVID-19 biology and identifies candidate biomarkers for improved diagnosis.

## Introduction

The coronavirus pandemic had a profound impact on the global economy and society [1]. Stringent socio-economic restrictions were implemented, and healthcare systems in many regions faced unprecedented strain [2]. As of August 2025, more than 778 million confirmed cases of Severe Acute Respiratory Syndrome Coronavirus 2 (SARS-CoV2) and more than 7 million deaths related to the infection have been reported worldwide [3].

COVID-19 can present with a high diversity of clinical manifestations, ranging from an asymptomatic course to severe inflammation-associated organ damage, mostly the lung, that requires intensive care unit (ICU) treatment [4,5]. Flu-like symptoms such as fever, dry cough, dyspnea, and myalgia are the most common symptoms. It was shown that around 81% of the cases were mild, 14% severe, and 5% critical [6]. In more severe cases, pneumonia and acute respiratory distress syndrome occur [7] and patients show a slow recovery process, especially in terms of lung function.

Beyond the acute phase manifestation of a SARS-CoV2 infection, it has become evident that a series of symptoms can occur or persist long after viral clearance. This phenomenon is termed long-COVID (or post-acute sequelae of COVID-19, chronic COVID syndrome, COVID-19 long hauler) [8] and affects about 2–20% of the infected individuals [9,10].

Although LC is widely recognized, the current literature emphasizes the lack of consensus regarding its definition, timelines, and clinical classification [11], as well as the difficulty of distinguishing it from recovery trajectories, with considerable overlap with other post-infectious syndromes. Importantly, very few studies have examined the transition zone between patients who recover from acute infection but still present with residual symptoms, and those who evolve into long-COVID. This highlights the need for studies that integrate clinical and biomarker-based approaches to achieve a clearer distinction [12,13]. For the present study, we adopted the World Health Organization (WHO)-led Delphi consensus as the definition for LC [14].

The pathomechanisms of severe COVID-19 and LC have been extensively investigated. Various hypotheses are discussed for the LC syndrome. A dysregulation of the immune response with metabolic dysregulation and endothelial damage may contribute to an inflammatory state and coagulation tendency [15], Persistent complement activation [16] and altered immunoglobulin signatures [17] have been linked to the LC phenotype, but little is known about the proteomic landscape of patients who are recovering from a severe COVID-19 infection without evolving into LC.

Our clinical experience at the Hochgebirgsklinik Davos provided the inspiration for the present study. In our rehabilitation setting, we routinely encounter two distinct groups: patients recovering shortly after a severe course of COVID-19, requiring pulmonary rehabilitation, and patients referred considerably later after mostly mild initial disease, presenting with persistent, heterogeneous symptoms typical of LC. While these two groups differ clearly in their clinical presentation, no study has systematically compared them within the same framework. To our knowledge, this is therefore the first study to introduce and characterize a PC group. This newly defined group offers the opportunity to distinguish between two post-infectious trajectories: one in which patients develop long-lasting, functionally limiting symptoms consistent with LC, and another in which patients, though weakened by the acute infection, are expected to recover over time.

Against this backdrop, the present study specifically aims to characterize and compare the proteomic immune profiles, clinical symptoms, and pulmonary function of patients with LC and those recovering from PC. To further refine group differentiation, we also applied a machine learning approach (Random Forest algorithm) to evaluate whether immunoproteomic signatures can predict and disentangle the two clinical trajectories. By integrating high-dimensional immunoproteomic analyses with clinical phenotyping, our overarching objective is to identify biomarkers and patterns that may help disentangle these trajectories, thereby advancing diagnostic precision and informing therapeutic strategies.

## Methods

### Study design, setting and participants

We conducted this monocentric observational study with a cross-sectional design at the Hochgebirgsklinik Davos, the largest rehabilitation clinic in the canton of Graubünden, Switzerland (1550 m above sea level), with a capacity of approximately 190 beds. The clinic closely collaborates with the University Hospital Zurich. The focus of the Hochgebirgsklinik is the transdisciplinary (Pneumology, Cardiology, Psychosomatics, Dermatology, Allergology/Immunology, Internal Medicine) treatment/rehabilitation of patients suffering from complex syndromes/ diseases entities. A focus among the latter are post-viral respiratory syndromes, chronic fatigue and LC. The Hochgebirgsklinik thus is a highly specialized center for the patient populations investigated in this study.

The study included participants with either LC (n = 24) or PC (n = 40) who were undergoing a rehabilitation stay at the clinic between May 2020 and April 2022. Individuals with only post-mild-COVID symptoms (pmC, n = 12) were included as a control group. The classification of PC, LC, and pmC was based on the time elapsed since the positive SARS-CoV-2 test and the medical indication for inpatient rehabilitation. Detailed criteria for these groups are provided in the Definitions section.

Inclusion criterium for all participants was a positive nasopharyngeal performed SARS-CoV-2 PCR or rapid test in the past (or a positive Anti-SARS-CoV2-IgG blood test for the pmC group). The inclusion criteria for each group differ in minor ways. Participants in the PC group were admitted for inpatient rehabilitation within 12 weeks of an acute infection with the virus. The LC group comprised individuals referred for inpatient rehabilitation due to persistent or new symptoms manifesting 12 weeks or more following the acute phase. Only participants who did not receive hospital treatment or special therapy during the acute infection were selected as subjects for the pmC group.

Exclusion criteria were age < 18 years, a positive SARS-CoV-2 test at inclusion, or inability to provide informed consent. 3 participants withdrew from participation before the first study visit.

Although three study visits were conducted during the rehabilitation stay: at admission (T0), after 2 weeks (T1), and at discharge (T2), i.e., 4–5 weeks after admission (S1 Fig), the present analysis includes only data collected at T0. Therefore, this manuscript represents a cross-sectional analysis comparing baseline characteristics across the three patient groups. The longitudinal analyses of T1 and T2 are reported separately.

### Data collection

Trained physicians interviewed participants at admission (T0) using a standardized questionnaire to document current and past symptoms, pre-existing medical conditions, medications, and treatments. Symptoms were recorded as dichotomous variables without further categorization of their severity.

#### Clinical examinations.

Clinical data on current and past symptoms, medications and treatment, and comorbidities were collected at T0 using a questionnaire. The symptoms were recorded as dichotomous variables without further categorization of their severity or specific manifestation. Pre-existing conditions were documented based on the participants’ medical diagnosis records.

At all three study visits, participants underwent a standardized physical examination including height, weight, temperature, blood pressure, heart rate, and auscultation of the lungs and heart. However, for the purposes of this manuscript, only the measurements from the initial assessment (T0) were analyzed.

#### Functional examinations.

At the T0 study visit, trained medical technical assistants (MTAs) performed a comprehensive set of functional assessments. These included lung function testing, diffusion capacity for carbon monoxide (DLCO), fractional exhaled nitric oxide (FeNO), blood gas analysis, electrocardiogram (ECG), and a 6-minute walk test (6MWT). A follow-up assessment (T1) repeated lung function, diffusion capacity for carbon monoxide, FeNO, and blood gas analysis. A final assessment (T2) at discharge incorporated another 6MWT and, for participants with adequate oxygenation without supplemental oxygen requirement, spiroergometry. For the present cross-sectional analysis, only the baseline measurements from T0 were used.

#### Biological sampling and processing.

Blood was collected from participants using coagulant-free tubes. All samples were processed immediately after collection. Serum was aliquoted and stored at −80°C until further analysis.

#### Blood and serum work-up.

Automated and differential blood counts were performed for all participants. Serum levels of tryptase, C-reactive protein (CRP), brain natriuretic peptide (NT-proBNP), Immunoglobulin E (IgE), D-dimers, troponin, lactate dehydrogenase (LDH), vitamin D, and Interleukin 6 (IL-6) were measured. All assays were performed according to the manufacturers’ instructions.

#### OLINK high-throughput targeted proteomics.

High-throughput targeted proteomics was performed using the OLINK target protein biomarkers system. Serum samples were tested with the OLINK multiplex assay (OLINK Target 96 Inflammation (92 protein biomarkers) and OLINK Target 96 Immune Response (92 protein biomarkers), OLINK Bioscience, Uppsala, Sweden) according to the manufacturer’s instructions. The incubation mix with oligonucleotide-labelled antibodies was incubated together with the samples. To increase specificity, each protein is labelled with two different epitope-specific antibodies. In the presence of the sought protein, the formation of a double-stranded oligonucleotide polymerase chain reaction (PCR) target is induced. For the detection of specific proteins, a dynamic array chip was primed, loaded with protein-specific primers and mixed with the samples including inter-plate controls and negative controls. Data were analysed using NPX (Normalised Protein Expression) managers. Data were normalised using internal controls in each sample, inter-plate normalisation controls and a correction factor. All samples were processed in a single batch [18]. In this study, OLINK technology was selected for its high sensitivity and specificity in detecting a broad range of protein biomarkers while requiring only small amounts of serum. Two selected multiplex assays (inflammation and immune response) were applied to investigate the inflammatory processes and potential ongoing immune responses following SARS-CoV-2 infection as previously described in studies applying these panels for COVID-19 and post-infection biomarker profiling [19,20]. The OLINK platform is predicated on the Proximity Extension Assay (PEA) technology, whereby pairs of antibodies linked to unique DNA oligonucleotides bind to their specific target proteins. When both antibodies bind in close proximity, their attached oligonucleotides hybridize and are subsequently amplified and quantified by real-time PCR. The dual recognition principle ensures high analytical specificity and sensitivity while allowing multiplex detection of up to 92 proteins per panel using minimal sample volumes [21].

### Definitions

LC was defined according to the WHO Delphi consensus definition of post-COVID-19 condition established in 2021: “Post-COVID-19 condition occurs in individuals with a history of probable or confirmed SARS-CoV-2 infection, usually 3 months from the onset of COVID-19 with symptoms that last for at least 2 months and cannot be explained by an alternative diagnosis. Common symptoms include fatigue, shortness of breath, and cognitive dysfunction, and generally have an impact on everyday functioning. Symptoms might be new onset after initial recovery from an acute COVID-19 episode or persist from the initial illness. Symptoms might also fluctuate or relapse over time.” [14] This definition has been widely adopted in reviews and large-scale studies investigating the condition [16,22,23]. The type and setting of treatment during the acute phase (outpatient, inpatient, or intensive care) were recorded but were not used to define this group.

PC was defined by the need for inpatient rehabilitation due to the severity of symptoms within 12 weeks after an acute COVID-19 infection. The key distinction for PC was the clinical requirement for rehabilitation, irrespective of the initial treatment setting (outpatient, inpatient, intensive care). Although microbiological confirmation of complete viral clearance after the acute infection was not available, participants were considered PC based on clinical recovery and subsequent referral to rehabilitation. This group represents a distinct post-infectious state characterized by a clinical trajectory of ongoing recovery which, while not aligning with the classical definition of LC, extends beyond the acute phase of the disease.

pmC participants were not hospitalized at the Hochgebirgsklinik Davos. When initially infected, they only had mild symptoms that did not persist. Participants in this group had either a positive SARS-CoV-2 PCR test or a positive anti-SARS-CoV-2 IgG (anti-spike) test. No hospitalization, specific treatment, or rehabilitation was required. This classification aligns with the WHO definition of mild COVID-19 infection [24].

### Ethical considerations

Each study participant was informed in detail and written consent for the study was obtained from all participants. At no point were the participants deprived of the opportunity to withdraw their participation in the study. Each participant was first assigned a unique identification number, and all personal data were anonymized to ensure confidentiality. Eligibility was determined solely based on predefined inclusion criteria, without any additional pre-selection. The treatment during rehabilitation did not differ from that of the non-participating patients.

The study was conducted in accordance with the principles of the Declaration of Helsinki and good clinical practice guidelines and approved by the ethics committees of Zürich under the approval number BASEC 2020−00898.

### Statistical analyses

Both traditional statistical analyses and machine learning analyses were performed to explore associations between clinical, laboratory, and proteomic variables. All analyses were conducted using IBM SPSS Statistics version 28 and R version 4.3.0 (Ubuntu v20.04.6).

Traditional statistical analyses were applied to clinical and laboratory variables, including demographic data, disease characteristics, and standard laboratory parameters.

Continuous, non-normally distributed variables were compared using the Mann–Whitney U test (for two groups) or the Kruskal–Wallis test (for multiple groups). Categorical variables were analyzed using the Chi-square test. These non-parametric tests were chosen because most variables did not meet assumptions of normal distribution and involved ordinal or continuous data. Multiple testing correction was performed using the Benjamini–Hochberg false discovery rate (FDR) method.

Proteomic data from OLINK analyses were processed using normalized protein expression (NPX) values. Group comparisons were performed with the Kruskal–Wallis test and Mann–Whitney U test using the “OlinkAnalyze” R package. The “limma” function from the OlinkR package was used for differential expression analyses, and FDR-adjusted p-values were reported. For visualization, the “ComplexHeatmap” package was used for heatmaps, and “EnhancedVolcano” for volcano plots. Summary statistics of clinical and laboratory data were generated using the R package arsenal (v3.6.3).

A random forest classification model was developed to differentiate LC from PC participants, following the TRIPOD-AI guidelines (see S2 Appendix in S2 Fig for the TRIPOD-AI checklist). The model was trained using the “caret” R package. The dataset was partitioned into training (80%) and test (20%) subsets using stratified sampling based on the outcome variable (condition) to maintain class balance. Model training and hyperparameter tuning were performed using 10-fold cross-validation with a single repetition. A total of 3,000 trees were grown to ensure model stability, and parallel processing (five worker nodes) was used to accelerate computation. A fixed random seed was applied to guarantee reproducibility.

Non-nested cross-validation was employed for model selection. Model performance was assessed exclusively on the held-out test set using accuracy, sensitivity, specificity, and area under the receiver operating characteristic curve (AUC).

Correlation analyses were performed on scaled data using pairwise complete observations. Pearson correlation coefficients were calculated, and p-values were adjusted using the Benjamini–Hochberg method. The resulting correlation matrix was visualized using the “corrplot” package, highlighting significant associations at predefined p-value thresholds (0.05, 0.01, 0.001).

Descriptive statistics for continuous variables are presented as median (interquartile range), and categorical variables as counts (percentages). Missing values were omitted. The OLINK dataset was complete; therefore, no imputation was necessary.

Graphical representations, including histograms, boxplots, dot plots, and heatmaps, were created using the “ggplot2” (v3.3.6) and “ComplexHeatmap” packages. Descriptive and inferential results are consistently presented in the Results section according to these visualization methods.

To ensure transparent and standardized reporting, the TRIPOD-AI checklist (S2 Fig) was followed for the machine learning analyses, and the STROBE checklist (S3 Fig) for observational studies.

## Results

### Baseline characteristics of participants

76 participants were included between 05/2020 and 04/2022. 40 participants had PC, 24 had LC, and 12 served as pmC controls. The median age of the PC group was 58.0 years, and 52.0 years for the LC group. There was a significantly higher proportion of female participants in the LC group (66.7%) in comparison to the PC group (30.0%, p = 0.006, Fishers Exact Test).

During the acute infection, 45.0% of PC participants were treated in the ICU and another 45.0% in non-ICU inpatient wards, while 10.0% were managed as outpatients. The majority (77.5%) received systemic glucocorticoids, 47.5% antibiotics, and 45.0% monoclonal antibodies (Tocilizumab, Casirivimab/Imdevimab). The mean time between the positive PCR test and begin of rehabilitation was 31 days. In contrast, LC participants were mostly outpatients during the acute infection, with only 29.2% hospitalized and 8.3% requiring ICU care. The mean time between COVID-19 diagnosis and admission was 45 weeks (range: 12–105 weeks).

There were significant differences in the treatment of LC and PC participants regarding the acute COVID-19 infection: 83.5% of the PC participants were treated with oxygen, whereas only 21.7% in the LC group (p-value <0.001 Fisher’s exact test). Overall, the proportion of invasive ventilation and non-invasive ventilation (NIV) was low in the LC group. In the PC group, invasive ventilation was performed in 30.0% and extracorporeal membrane oxygenation (ECMO) therapy was necessary in 3 participants (7.5%).

There were no significant differences between the groups in terms of current smokers (12.5% vs. 20.0%; p = 0.53), ever having smoked (37.5% vs. 40.0%; p = 1.0) or pre-existing lung disease (37.5% vs. 20.0%; p = 0.15, Fisher’s exact test) (Table 1).

**Table 1:** Demographic and clinical characteristics, hospitalization course, and acute-phase therapies in LC (n = 24), PC (n = 40) and pmC (n = 12) cohorts at baseline (T0). Group comparisons were performed using Chi-square, Kruskal–Wallis tests, Mann Whitney-U and Fisher`s exact test.

	Long-COVID/LC (n = 24)	Post-severe COVID/PC (n = 40)	Post-mild COVID/pmC (n = 12)	All (n = 76)	p-values
**Demographics**					
Male, n (%), 95% CI	8 (33.3%), 18–53.3%	28 (70.0%), 54.6–81.9%	5 (41.7%), 19.3–68%	41 (54.0%)	**0.006**
Female, n (%), 95% CI	16 (66.7%), 46.7–82%	12 (30.0%), 18.1–45.4%	7 (58.3%), 32–80.7%	35 (46.0%)	**0.006**
**Age**, median (IQR)	52.0 (37.0; 60.25)	58.0 (48.0; 65.0)	42.0 (28.0; 52.0)	56.0 (42.5; 63.5)	0.011
**BMI**, median (IQR)	30.45 (25.93; 33.60)	26.85 (23.70; 29.80)	NA	27.85 (24.50; 31.30)	**0.0023**
Current smoker, n (%), 95% CI	3 (12.5%), 4.4–31%	8 (20%), 10.5–34.8%	NA	11 (14.5%)	0.53
Ever smoked, n (%), 95% CI	9 (37.5%), 21.7–56.4%	16 (40%), 26.4–55.4	NA	25 (32.9%)	1.0
Preexisting lung disease^1^, n (%), 95% CI	9 (37.5%), 21.7–56.4	8 (20%), 10.5–34.8	NA	17 (22.4%)	0.15
**Hospitalization**					
ICU, n (%), 95% CI	2 (8.3%), 2.3–25.8	18 (45.0%), 30.7–60.2	0 (0%)	20 (31.2%)	**<0.001**
Hospitalization without ICU, n (%), 95% CI	7 (29.2%), 14.9–49.2	18 (45.0%), 30.7–60.2	0 (0%)	25 (39.1%)	**0.023**
**ICU treatment: Ventilation**					
Oxygen, n (%), 95% CI	5 (21.7%), 9.7–41.9	33 (82.5%), 68.1–91.3	0 (0%)	38 (60.3%)	**<0.001**
Highflow, n (%), 95% CI	1 (4.2%), 0.7–20.6	18 (46.2%), 31.7–61.4	0 (0%)	19 (30.2%)	**<0.001**
Invasive ventilation, n (%), 95% CI	2 (8.3%), 2.3–25.8	12 (30.0%), 18.0–45.9	0 (0%)	14 (21.9%)	**0.034**
Prone position, n (%), 95% CI	2 (8.3%), 2.3–25.8	7 (17.5%), 8.8–31.6	0 (0%)	9 (14.1%)	0.344
ECMO, n (%), 95% CI	0 (0.0%), 0–13.8	3 (7.5%), 2.5–20.2	0 (0%)	3 (4.7%)	0.618
**Medications during acute phase**					
Systemic glucocorticosteroids, n (%), 95% CI	5 (20.8%), 9.2–40.4	31 (77.5%), 62.5–87.8	0 (0%)	36 (56.2%)	**<0.001**
Inhalative glucocorticosteroids, n (%), 95% CI	3 (12.5%), 4.4–31.0	1 (2.5%), 0.4–13.2	0 (0%)	4 (6.2%)	0.246
Antibiotics ^2^, n (%), 95% CI	3 (12.5%), 4.4–31.0	19 (47.5%), 32.5–62.9	0 (0%)	22 (34.4%)	**<0.001**
Anticoagulation^3^, n (%), 95% CI	1 (4.2%), 0.7–20.6	9 (22.5%), 12.1–37.9	0 (0%)	10 (15.6%)	0.065
Chloroquine/Remdesivir, n (%), 95% CI	1 (4.2%), 0.7–20.6	2 (5.0%), 1.4–16.5	0 (0%)	3 (4.7%)	0.983
Monoclonal antibodies^4^, n (%), 95% CI	1 (4.2%), 0.7–20.6	18 (45.0%), 30.7–60.2	0 (0%)	19 (29.7%)	**<0.001**

^1^Asthma/chronic obstructive lung disease/restrictive lung disease.

^2^Amoxicillin/Clavulanic acid, Piperacillin-Tazobactam, Ceftriaxon.

^3^Marcoumar, Rivaroxaban, Heparin.

^4^Tocilizumab, Casirivimab/Imdevimab.

Abbreviations: BMI (body mass index), ICU (intensive care unit), ECMO (extracorporeal membrane oxygenation), NA (not available).

### Clinical profiles of LC and PC participants

At the time of admission to rehabilitation, LC participants frequently reported fatigue (58.3%), dyspnea (50.0%), sleep disturbances (50.0%), concentration problems (41.7%), and muscle weakness (33.3%), whereas PC participants presented predominantly with respiratory symptoms, including dyspnea (82.5%) and cough (45.0%) (Table 2). Compared to PC, LC participants showed a significantly higher prevalence of fatigue, sleep disorders, muscle weakness, concentration difficulties, and neurological complaints (all p < 0.05).

**Table 2:** Symptom burden in LC (n = 24) and PC (n = 40) participants assessed at the beginning of rehabilitation (T0). Group comparisons were performed using the Chi-square and Wilson test.

Symptom	Long COVID/LC(n = 24)	Post-severe COVID/PC (n = 40)	Total (n = 64)	p value
**Respiratory symptoms**				
Cough, n (%), 95% CI	5 (20.8%), 9.2-40.2%	18 (45.0%), 31.1-39.7%	23 (35.9%)	0.051
Sputum, n (%), 95% CI	2 (8.3%), 2.3-25.8%	6 (15.0%), 7.1-28.9%	8 (12.5%)	0.435
Dyspnea, n (%), 95% CI	12 (50.0%), 31.6-68.4%	33 (82.5%), 68.2-91.3%	45 (70.3%)	**0.006**
Sniffles, n (%), 95% CI	1 (4.2%), 0.7-21.1%	0 (0.0%), 0-8.8%	1 (1.6%)	0.193
**General symptoms**				
Fever, n (%), 95% CI	4 (16.7%), 6.7-35.9%	2 (5.0%), 1.4-16.5%	6 (9.4%)	0.121
Fatigue, n (%), 95% CI	14 (58.3%), 38.8-75.5%	5 (12.5%), 5.6-25.7%	19 (29.7%)	**< 0.001**
Muscle atrophy, n (%), 95% CI	8 (33.3%), 18-53.3%	4 (10.0%), 4-23.1%	12 (18.8%)	**0.021**
Sleep disorders, n (%), 95% CI	12 (50.0%), 31.6-68.4%	6 (15.0%), 7.1-28.9%	18 (28.1%)	**0.003**
**Gastrointestinal symptoms**				
Loss of smell/taste, n (%), 95% CI	5 (20.8%), 9.2-40.2%	3 (7.5%), 2.6-20.1%	8 (12.5%)	0.118
Diarrhea, n (%), 95% CI	1 (4.2%), 0.7-21.1%	1 (2.5%), 0.4-15.1%	2 (3.1%)	0.711
Nausea/emesis, n (%), 95% CI	2 (8.3%), 2.3-25.8%	0 (0.0%), 0-8.8%	2 (3.1%)	0.064
**Neurological symptoms**				
Headache, n (%), 95% CI	5 (20.8%), 9.2-40.2%	4 (10.0%), 4-23.1%	9 (14.1%)	0.227
Concentration issues, n (%), 95% CI	10 (41.7%), 24.5-61.2%	3 (7.5%), 2.6-20.1%	13 (20.3%)	**0.001**
Neurological issues, n (%), 95% CI	6 (25.0%), 12.1-44.9%	1 (2.5%), 0.4-15.1%	7 (10.9%)	**0.005**
**Cardiovascular symptoms**				
Heart problems, n (%), 95% CI	4 (16.7%), 6.7-35.9%	0 (0.0%), 0-8.8%	4 (6.2%)	**0.008**
Performance intolerance, n (%), 95% CI	5 (20.8%), 9.2-40.2%	10 (25.0%), 14.6-39.6%	15 (23.4%)	0.703

### Lung function in PC and LC participants

**Fig 1:**
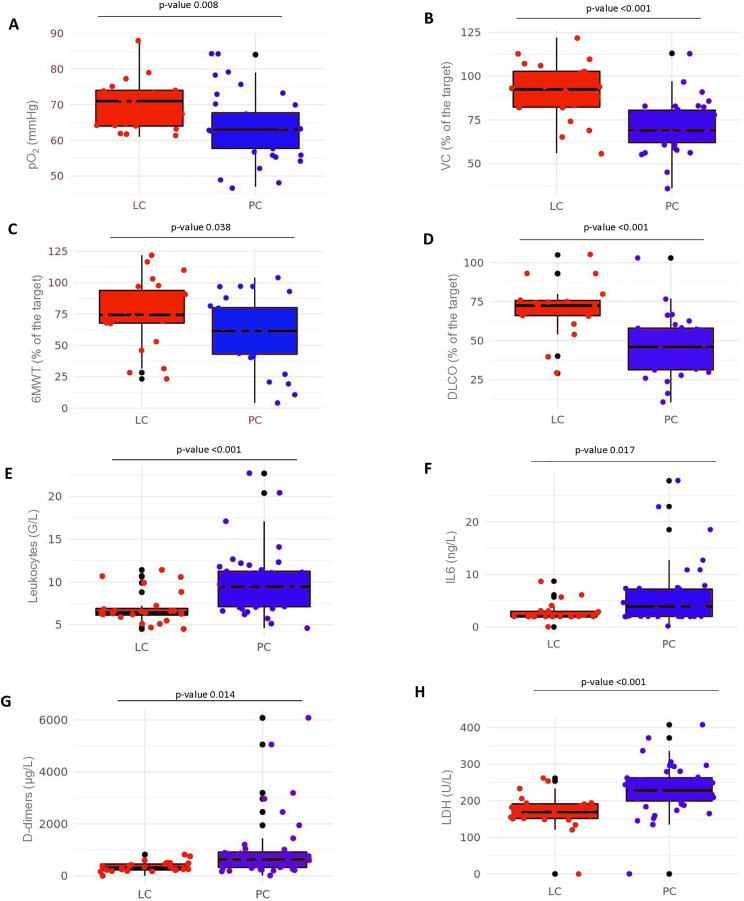
Lung function parameters and standard laboratory parameters at T0 comparing LC and PC participants. At the timepoint T0 lung function examination and routine lab was performed in LC and PC participants and depicted in box plots including p-value. The box plots show the median including Q1 and Q3 **(A)** partial pressure of oxygen (pO2) measured in capillary blood from the ear. Normal value at 1500m: > 60mmHg **(B)** Vital capacity of the lung (VC) measured by body plethysmography. Normal value >80% of population-specific target value (measured by age, sex, height and weight). **(C)** 6-minute walk test (6MWT) carried out on the level at 1500m. Normal value >80% of the population-specific target value **(D)** Diffusing capacity of the lung for carbon monoxide (DLCO) was measured by standard diffusion testing. The corresponding reference values were calculated using established prediction equations, which account for demographic and anthropometric variables including age, sex, height, and weight. Normal values were defined as >80% of the population-specific predicted value derived from these equations [25] **(E)** Leukocytes in the unit G/l (10^9^/L). Normal value 4–10 x10^9^/ L. A complete blood count was performed. **(F)** Interleukin-6 (IL-6) in the unit ng/L. Normal value <2,0 ng/L. Laboratory chemistry did not allow analysis of values below 2.0ng/L. A result <2.0ng/L was entered as 2.0ng/L. **(G)** D-dimers in the unit µg/L. Normal value <500 µg/L. **(H)** Lactate dehydrogenase (LDH) in the unit U/L. Normal value < 232U/L. Statistical test: Mann-Whitney U test.

Pulmonary function testing revealed significant impairment in PC compared to LC participants. The median oxygen partial pressure (pO₂) in PC was 63.0 mmHg vs. 71.0 mmHg in LC (p = 0.008). Vital capacity and diffusion capacity of carbon monoxide were markedly reduced in PC (vital capacity/VC = 69.0% vs. 92.5%; diffusion capacity/DLCO = 46.0% vs. 72.5%; both p < 0.001). The 6-minute-walk test as a marker of cardiovascular and pulmonary performance below the anaerobic threshold was significantly reduced in the PC group (6MWT median: 61.50% of the target vs. 74.43% of the target, p = 0.038; norm >80% of the target) (Figs 1 and S4, Table 3).

**Table 3:** Pulmonary function parameters and functional exercise capacity in LC (n = 24) and PC (n = 40) participants at baseline (T0). Group comparisons were performed using the Mann-Whitney U test.

Parameter	Long-COVID/LC (n = 24)	Post-severe COVID/PC (n = 40)	Total (n = 64)	p value
Partial pressure of oxygen - pO_2_ (mmHg), Median (IQR)	71.00 (64.00, 74.00)	63.00 (57.75, 67.75)	64.00 (59.75, 73.00)	**0.008**
Vital capacity – VC (% of the target), Median (IQR)	92.50 (82.25, 102.75)	69.00 (62.00, 80.50)	78.50 (67.75, 91.25)	**<0.001**
Forced expiratory capacity 1 sec. - FEV1 (% of the target), Median (IQR)	84.50 (71.50, 93.00)	71.00 (60.00, 81.00)	76.00 (64.00, 88.00)	0.128
Diffusing capacity for carbon monoxide – DLCO (% of the target), Median (IQR)	72.50 (66.00, 75.75)	46.00 (31.25, 58.00)	58.00 (39.75, 72.25)	**<0.001**
Exhaled NO – FeNO (ppmb), Median (IQR)	25.20 (16.40, 30.40)	16.95 (10.47, 28.70)	23.00 (12.30, 30.00)	0.24
6 minutes of walking – 6MWT (% of the target), Median (IQR)	74.43 (67.78, 93.82)	61.50 (42.98, 80.11)	69.34 (46.37, 88.47)	**0.038**

### Distinct systemic inflammation profiles in PC vs. LC

We were then interested in the cellular and molecular indicators of systemic inflammation and whether they would parallel the distinct manifestations of PC vs. LC. The complete differential blood count showed an increase of overall leukocytes (median PC: 9.45 vs. median LC: 6.40 G/l), but no significant differences in lymphocytes (median PC: 2.49G/L vs. LC: 2.32G/L p = 0.701), eosinophils (p = 0.376) or basophils (p = 0.688). This increase was paralleled by higher Interleukin 6 levels (IL-6 median: PC: 3.9 vs. LC: 2.0 ng/l, p = 0.017) and an increase in coagulation parameters (d-dimers median: PC: 633.5 vs. LC: 312.0 ug/l, p = 0.014). The level of CRP, one of the most important inflammatory parameters, was only slightly elevated in the PC group, and normal in the LC group (median PC: 4.65 mg/L vs. LC: 2.40 mg/L, p = 0.283) (Figs 1 and S5, Table 4).

**Table 4:** Laboratory assessment of inflammatory markers, coagulation parameters, and cardiac biomarkers in LC (n = 24) and PC (n = 40) participants at baseline (T0). Group comparisons were performed using the Mann-Whitney U test.

Parameter	Long-COVID/LC (n = 24)	Post-severe COVID/PC (n = 40)	Total (n = 64)	p value
Leukocytes (G/L), Median (IQR)	6.40 (6.12, 6.90)	9.45 (7.10, 11.25)	7.70 (6.40, 10.77)	**<0.001**
CRP/C-reactive Protein (mg/L), Median (IQR)	2.40 (0.47, 5.42)	4.65 (1.10, 8.70)	3.80 (0.82, 8.20)	0.283
NT-proBNP/brain natriuretic peptide (ng/L), Median (IQR)	34.50 (27.25, 45.00)	58.00 (29.75, 149.25)	41.00 (29.00, 93.75)	0.103
IgE/Immunoglobulin E (kU/L), Median (IQR)	39.00 (9.95, 123.00)	35.20 (9.56, 103.00)	36.20 (9.65, 104.50)	0.999
D-Dimer (ug/L), Median (IQR)	312.00 (225.00, 455.50)	633.50 (324.25, 916.75)	432.50 (253.00, 762.00)	**0.014**
LDH/lactate dehydrogenase(U/L), Median (IQR)	169.00 (151.75, 191.75)	228.50 (199.25, 263.25)	206.00 (165.75, 249.50)	**<0.001**
IL6/interleukin 6 (ng/L), Median (IQR)	2.00 (2.00, 3.00)	3.90 (2.00, 7.22)	2.90 (2.00, 5.75)	**0.017**

**Fig 2:**
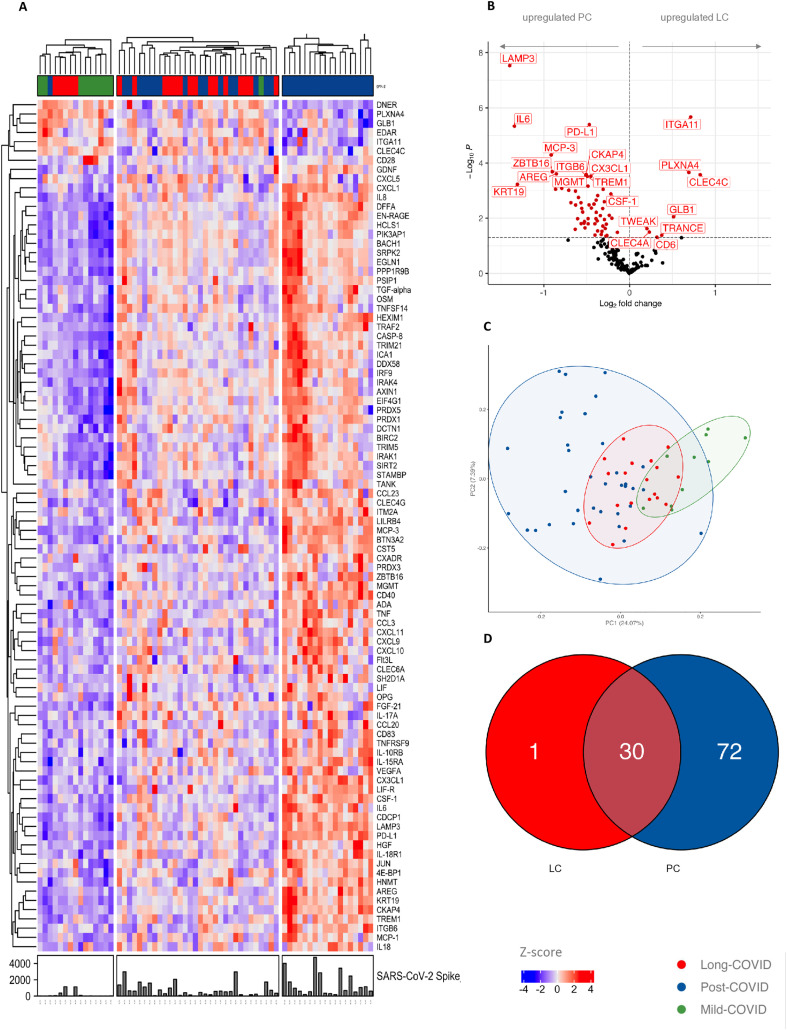
Heatmap of OLINK proteins (inflammation panel and immune response panel) in LC, PC and pmC participants. **(A)** Depicted is an unsupervised clustering of OLINK proteins in 3 groups. Forming a group of only PC participants (right), a group of mixed LC and PC participants (middle) and a group of pmC and LC participants (left). **(B)** Volcano plot with upregulated OLINK proteins of the groups LC and PC. **(C)** Principal component analysis of the three groups LC, PC and pmC. **(D)** Venn diagram of the upregulated OLINK proteins in the LC and PC groups. The diagram shows the number of upregulated OLINK proteins in the respective groups. In the LC group 31 proteins are upregulated, in the PC group 102. 30 of them are upregulated in both groups.

We further investigated the systemic molecular signatures of inflammation in PC and LC respectively using high-throughput targeted proteomics. A comparison of PC, LC, and pmC participant groups revealed major differences. LC and PC tended to cluster separately from pmC as depicted in the principal component analysis (PCA) (Fig 2C). 92 of the 180 analyzed proteins were differentially expressed between the three groups. An unsupervised clustering containing proteins with significantly different expression (Fig 2A) showed a distinct grouping. Based on the clustering, we could distinguish three groups: one exclusively with PC participants, one with PC and LC participants, and one group with LC and pmC participants. The analysis of the baseline data in this last group, collected at time T0 on pre-existing conditions, medication and the course of acute SARS-CoV-2 disease, showed pmC participants and LC participants with hardly any comorbidities, a mild course of COVID-19 disease, and no regular medication intake.

**Fig 3:**
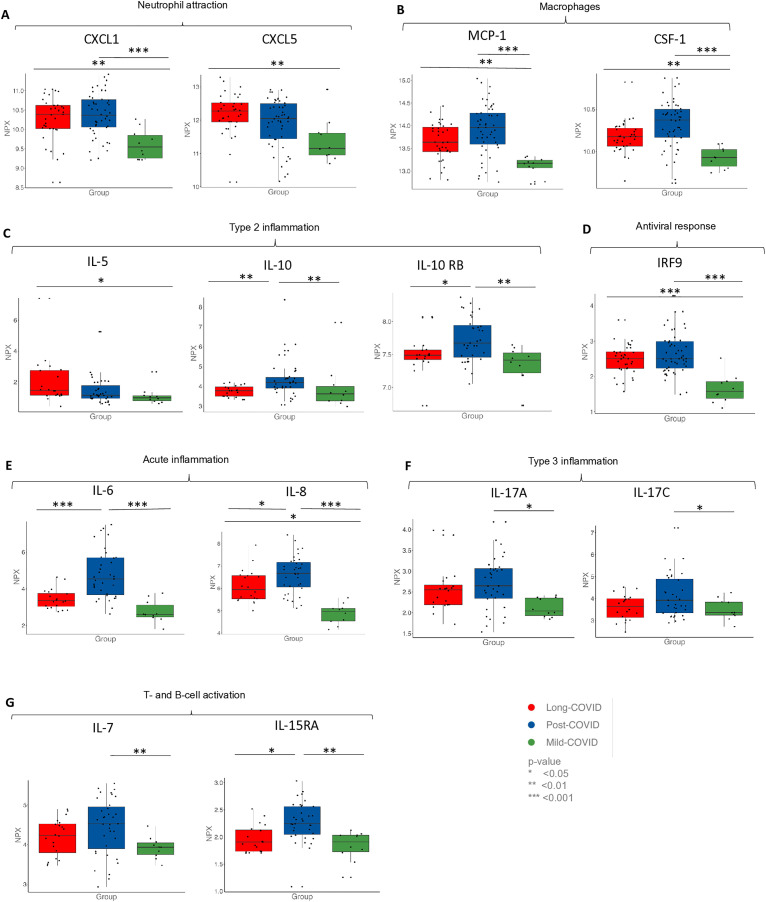
OLINK proteins with a regulatory function in inflammation, comparing LC, PC and pmC participants at timepoint T0. Depicted are boxplots representing three different participant groups. Each boxplot illustrates the median as well as the first (Q1) and third quartiles (Q3). Statistical significance between groups is indicated by asterisks (* p < 0.05; ** p < 0.01; *** p < 0.001). The y-axis displays NPX (Normalized Protein Expression) values on a log2 scale, representing relative protein abundance. **(A)** Chemokine ligand 1 + 5 (CXCL1 and CXCL5), both proteins in the pathway of attraction of neutrophils **(B)** Colony stimulating factor 1 (CSF-1) and Monocyte Chemoattractant Protein 1 (MCP-1) are both important proteins in recruiting of macrophages **(C)** Interleukin 5, 10 and Interleukin 10 receptor subunit beta (IL-5, IL-10, IL-10 RB) are chemokins in type 2 Inflammation **(D)** Interferon regulatory factor 9 (IRF-9) plays a role in the antiviral response **(E)** Interleukin 6 + 8 (IL-6 IL-8) play a role in acute Inflammation **(F)** Interleukin 17 A + C (IL17A, IL17C) are cytokins in type 3 inflammation **(G)** Interleukin 7 and Interleukin 15 receptor subunit alpha (IL7, IL15RA) play a role in T- and B-cell activation.

Regarding the possible involvement of immune activation pathways, our OLINK data showed a significantly different distribution in PC, LC and pmC. The neutrophil chemoattractants, Chemokine ligand 1 + 5 (CXCL1 and CXCL5) and macrophage activation-proteins (Monocyte Chemoattractant Protein 1/MCP-1, Colony stimulating factor 1/CSF-1) were increased in LC and PC compared to pmC (Fig 3A/B).

### Proteomic signatures in PC and LC and their clinical correlates

A comparison of the upregulated proteins in PC and LC showed that Lysosome-associated membrane glycoprotein 3 (LAMP3) was the most upregulated protein in PC. In line with the routine laboratory work-up, IL-6 and other proinflammatory cytokines such as MCP-1 and CSF-1 were increased in PC participants. Immune inhibitory process-related proteins such as programmed cell death 1 ligand 1 (PD-L1) were also upregulated. In the LC group, the most upregulated proteins included fibrotic as well as dendritic cell markers such as integrin alpha-11 (ITGA11), Plexin A4 (PLXNA4), and c-type lectin domain family 4 member A and C (CLEC4C, CLEC4A) (Fig 2B).

**Fig 4:**
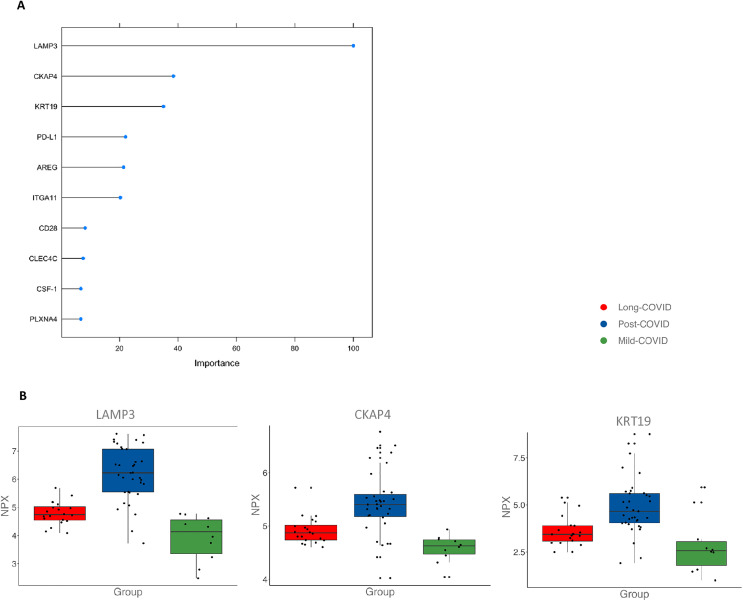
Parameters to distinguish PC from LC. **(A)** To distinguish PC from LC participants a machine learning algorithm was applied. The 10 most important parameters out of all 184 OLINK parameters and their respective significance are shown in the bar chart. **(B)** The three most important parameters from the machine learning algorithm - LAMP3 (Lysosome-associated membrane glycoprotein 3), KRT19 (Keratin 19), and CKAP4 (Cytoskeleton-associated protein 4) – are depicted as boxplots for the three groups PC, LC, and pmC. Boxplots display the median, first (Q1) and third quartiles (Q3). The y-axis shows NPX (Normalized Protein Expression) values on a log2 scale, indicating relative protein abundance. Statistical significance is indicated by asterisks (* p < 0.05; ** p < 0.01; *** p < 0.001).

To elaborate a potential biomarker profile that enables the differentiation of LC and PC participants based solely on laboratory parameters without the use of clinical parameters, a random forest algorithm was trained. With an accuracy of around 89%, it was possible to differentiate LC and PC based on the panel of 184 proteins. Interestingly, calculating variable importance for our model shows that LAMP3 plays a significant role, while cytoskeleton associated protein 4 (CKAP4) and Keratin 19 (KRT19) are moderately important. This is a strong indication that these features are highly influential in our Random Forest model, potentially leading to clinical evaluation (Fig 4A/B).

In the group of LC participants, the clinical and laboratory parameters were inconspicuous compared to the PC participants. However, this group showed specific symptoms such as fatigue and brainfog, which were not described by the participants in the PC and pmC group. However, we did not observe any clear association between these symptoms and specific OLINK protein levels (S6 FigA/B).

The body mass index (BMI) of the two groups was increased on average with a higher BMI in the LC group (29.2 vs. 28.1 kg/m²; normal range 18.5–25 kg/m²). Among the tested protein biomarkers, a significant correlation was identified only between BMI < 30 and elevated levels of integrin beta 6 (ITGB6) (S6 Fig).

We generated a Pearson correlation matrix to explore potential relationships between (i) key routine laboratory parameters, (ii) selected clinical parameters, (iii) the time interval between COVID-19 diagnosis and serum sampling, and (iv) OLINK protein levels. This analysis revealed a significant correlation between reduced diffusion capacity (DLCO) and several OLINK parameters (S7 Fig).

**Fig 5:**
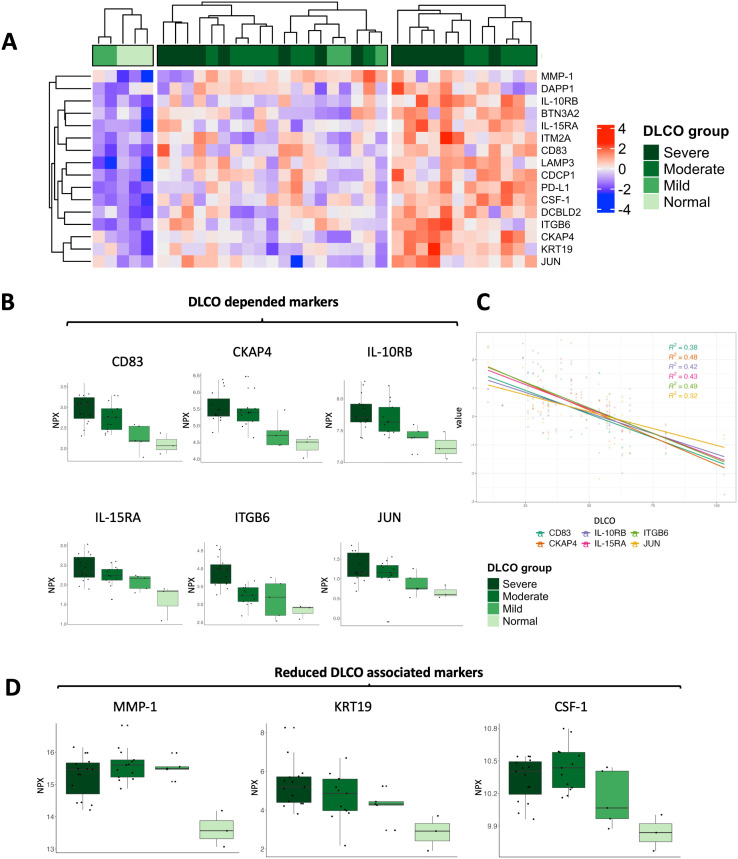
Relationship between diffusion capacity and OLINK proteins. An analysis of clinical parameters, routine laboratory data, and OLINK proteins at timepoint T0 revealed a consistent relationship between reduced diffusion capacity and the upregulation of several OLINK proteins. **(A)** Heatmap of selected OLINK proteins grouped into three distinct clusters. The top annotation indicates the respective category of diffusing capacity of the lung for carbon monoxide (DLCO), which was measured as a percentage of the predicted value [25]. Patients were stratified into four DLCO groups: normal (>80%)**,** mildly reduced (60–80%)**,** moderately reduced (40–60%), and severely reduced (<40%) **(B)** Boxplots of OLINK proteins showing levels proportional to the severity of diffusion impairment: cluster of differentiation 83 (CD83); cytoskeleton-associated protein 4 (CKAP4); interleukin-10 receptor subunit beta (IL-10RB); interleukin-15 receptor subunit alpha (IL-15RA); integrin beta 6 (ITGB6); and transcription factor AP-1 (JUN). **(C)** Six linear regression plots with the according Pearson correlation coefficient illustrating the negative association between DLCO values and the expression levels of selected OLINK proteins from panel **(B)**: CD83, CKAP4, IL-10RB, IL-15RA, ITGB6, and JUN. **(D)** Boxplots of additional OLINK proteins associated with reduced diffusion capacity: matrix metalloproteinase-1 (MMP-1); keratin 19 (KRT19); and colony stimulating factor 1 (CSF-1).

To further investigate this connection, we grouped the PC participants into four categories based on their DLCO impairment. They ranged from normal (DLCO >80% of the target) to severely impaired DLCO (DLCO <40% of the target). We found a clear positive correlation between levels of cluster of differentiation 83 (CD83), cytoskeleton associated protein 4 (CKAP4), interleukin 10 receptor subunit beta (IL-10RB), interleukin 15 receptor subunit alpha (IL-15RA), integrin beta 6 (ITGB6), transcription factor AP-1 (JUN) and decreased DLCO, i.e., worsened lung function (Fig 5A/B/C). In addition, three proteins from the OLINK analysis showed a positive association with reduced diffusion capacity, namely matrix metalloproteinase-1 (MMP-1); keratin 19 (KRT19); colony stimulating factor 1 (CSF-1) (Fig 5D).

Although data were collected at multiple time points, the present study did not include an analysis of longitudinal changes over time. The focus was on cross-sectional comparisons between the PC, LC, and pmC groups rather than evaluating temporal trends in biomarkers or functional tests.

## Discussion

This study investigated clinical, pulmonary, and proteomic characteristics of participants with post-severe PC and LC. The main findings were: (i) PC and LC represent two distinct post-acute phenotypes; (ii) PC participants showed persistent pulmonary restriction and inflammatory activity; (iii) LC participants displayed systemic and neurocognitive symptoms with distinct proteomic profiles; (iv) several inflammatory and fibrotic proteins were associated with impaired lung diffusion; and (v) machine learning identified discriminatory biomarkers distinguishing PC and LC with high accuracy.

Clinical features differed markedly between groups. LC participants reported fatigue, cognitive impairment, and sleep disorders as predominant symptoms, whereas PC participants primarily exhibited respiratory limitations. These findings indicate different organ system involvement and symptom constellations during post-acute recovery.

In PC participants, we detected, even about 30 days post-infection, considerable lung function impairment. This was demonstrated by reduced lung function, diffusion, oxygenation and aerobic capacity. It is consistent with previous studies of severe COVID-19 with prolonged recovery and highlights the importance of pulmonary rehabilitation [26,27]. There was no significant deterioration in lung function parameters in the LC group. However, participants reported a variety of symptoms, including fatigue, neurological symptoms, sleep problems and difficulty concentrating in addition to the classic COVID-19 symptoms of dyspnea and cough. A similar picture emerges from various studies, which describe a wide range of heterogeneous symptoms in LC patients [14,28].

Routine laboratory marker analysis showed increased inflammation, coagulation activation and cardiac involvement, particularly D-dimer and NT-proBNP. D-dimers are a commonly used marker of thrombosis, but are also elevated in systemic inflammatory responses and are detectable up to 4 months after SARS-CoV2 infection [29]. NT-proBNP is released during ventricular dilatation and indicates direct or indirect cardiac stress. Studies have shown that elevated NT-proBNP in COVID-19 is associated with increased 30-day mortality [30].

In the PC group, besides proinflammatory cytokines, Lysosome-associated membrane glycoprotein 3 (LAMP3) was found to be upregulated. LAMP3 is predominantly expressed in dendritic cells and has been implicated in immune regulation. High LAMP3 expression has been associated with T cell activation and chemokine signaling pathways [31]. Programmed cell death ligand 1 (PD-L1) was upregulated, which is in line with previous studies suggesting it as a severity marker of COVID-19 [32,33].

Only a few immune response-related proteins were overexpressed in LC compared to PC group: integrin alpha-11 (ITGA11), Plexin A4 (PLXNA4), and c-type lectin domain family 4 member (CLEC4). ITGA11 is a profibrogenic membrane protein [34] expressed by myofibroblasts in fibrotic diseases. The ITGA11 elevation in LC participants could be due to a mild pulmonary fibrosis. Although the risk of pulmonary fibrosis during convalescence after SARS-CoV-2 infection has been investigated in numerous studies [35], our study is the first to evoke ITGA11 as a possible biomarker and pathogenic mediator. Plexin A4 (PLEXNA4) is involved in the release of cytokines induced by Toll-like receptors and is important for regulating immune cell interactions, such as activation, differentiation and mobilisation, via the semaphorin-plexin signalling pathway [36].

The high PLEXNA4 levels in LC participants could indicate a persistent, increased reactivity to certain inflammation. CLEC4, also known as BDCA2 or dendritic lectin, is a membrane protein of plasmacytoid dendritic cells that can inhibit interferon-expression (IFN-a/ IFN-b [37,38]. Previous studies hypothesized that IFN-a and -b dysregulation underlies long-COVID. Our results however would suggest a compensatory over-expression of CLEC4 to counteract IFN overexpression in LC [35].

In addition, we were able to detect an association between a reduced diffusion capacity (DLCO) and various biomarkers. In particular, proteins with a regulatory function in inflammation (cluster of differentiation 83/CD83, colony stimulating factor/CSF-1, interleukin 10/IL-10, interleukin 15/IL-15, transcription factor AP-1/JUN) and proteins with a fibrosing effect (matrix metalloproteinase-1/MMP-1, integrin beta 6/ITGB6) stand out. Interleukin-10 is generally considered to be a regulator of inflammation. However, previous studies have found increased levels of IL-10 in COVID-19 patients and correlated it with increased mortality [39]. We found increased pro-inflammatory parameters in the PC group. This supports the hypotheses of IL-10 resistance [40,41] or a pro-inflammatory effect of IL-10 [34]. ITGB6 was shown in the study by Bowman et al to be an important component of proteomic signature in interstitial lung disease [42]. MMP-1 is a collagenase that breaks down the extracellular matrix. It is discussed as one of the mediators of pulmonary injury in COVID-19 and as one of the prognostic factors during the acute Infection [43–45].

A distinction between PC and LC could be made using the protein biomarkers LAMP3, CKAP4 and KRT19 in a random forest classifier with an accuracy of 89%. Interestingly, KRT19 has already been identified in several studies as an important biomarker for predicting the severity of COVID-19 infection [46–48].

Various studies have been conducted on the pathogenesis of long COVID syndrome. Several hypotheses are currently being discussed [49]. Among others, an autoimmunity via molecular mimicry [50] and a transient production of autoantibodies during acute SARS-CoV-2 [51] as well as a dysregulation of the immune system [52–55] are discussed. In addition, there is the hypothesis of persistent reservoirs for SARS-CoV-2 [56,57], an effect on the microbiome and virome [58], as well as effects on microvascular blood coagulation with endothelial dysfunction [59]. A recent study by Cervia-Hasler, Boyman et al. demonstrated an increased and persistent activation of the complement system with a thromboinflammatory signature in patients with LC symptoms [16]. In our study, no direct markers of the complement system were determined, but there was also an increase in proinflammatory markers in both the PC and LC groups as well as an increase in prothrombogenic markers, but only in the group of PC participants with an increase in coagulation parameters.

## Limitations

The recruitment period of our study was about two years. During this period, different variants of the coronavirus were detected worldwide and in Switzerland [60,61]. However, the present study did not use genome sequencing to identify the different variants, and therefore, the potential effects of different variants could not be accounted for in the analysis. However, there were only few changes within the groups during the recruitment period of two years. Drugs such as cortisone and monoclonal antibodies tended to be used more frequently towards the end of the study period, especially in the PC group. Vaccination against SARS-CoV2 only became available during the course of the study. This has resulted in groups that were heterogenous in terms of their vaccination status. As we did not conduct a follow-up beyond the rehabilitation period, we cannot rule out that some PC patients subsequently developed persistent symptoms meeting the criteria for LC.

Participants’ symptoms were recorded dichotomously using a questionnaire. However, standardized assessment tools that allow for a more detailed evaluation of symptom severity, such as the Modified Medical Research Council (mMRC) Dyspnea Scale or the St. George’s Respiratory Questionnaire (SGRQ) for dyspnea, were not implemented. As a result, this study is unable to differentiate between mild and severe symptom manifestations.

The study population was pre-selected based on referral for inpatient rehabilitation, a factor beyond the influence of the study management. Secondly, the timing of blood sampling was not standardized relative to the initial infection or symptom onset. Samples were drawn at the start of rehabilitation, meaning the interval varied considerably between groups. Moreover, for participants classified as PC, microbiological confirmation of complete viral clearance after the acute infection was not available. This pre-selection and lack of virological confirmation limit the generalizability of the findings to the broader population.

The LC and PC groups were defined primarily by the time since acute infection and clinical referral pattern. This resulted in cohorts with a mixture of initial severe cases of the disease. The inherent link between the initial illness severity and the subsequent post-acute syndrome means that residual confounding cannot be entirely ruled out. Consequently, the findings should be interpreted as characterising distinct clinical presentations that are influenced by both the timing post-infection and the initial disease severity.

The study was conducted as a monocentric investigation at a rehabilitation clinic in Davos, with a relatively small sample size.

The present study did not employ a pre-specified sample size calculation; rather, it utilised a convenience sampling method, including all eligible patients during the study period. While this was a necessary approach for this pioneering investigation during the uncertain landscape of the pandemic, it may impact the generalizability of our findings and the precision of the estimates. The resulting sample size, particularly for the LC group (n = 24), also limited the complexity of the statistical models that could be reliably fitted.

Additionally, the sample sizes of the study groups varied, with the pmC group being relatively small. Since this group primarily served as a comparison for mild COVID-19 cases, its sample size was determined based on statistical power calculations specific to the study’s main research question.

## Conclusion

This study provides robust biological evidence that PC and LC represent distinct clinical entities driven by different immune responses. We identified distinct immune signatures, characterized by elevated inflammatory and pro-fibrotic proteins in PC participants, which differentiate these conditions and suggest a key role for persistent inflammation and fibrotic processes.

Critically, we demonstrate that these signatures have direct clinical utility. A random forest classification algorithm effectively distinguished PC from LC with high accuracy, highlighting key biomarkers like LAMP3, KRT19, and CKAP4.

While the heterogeneity of long-COVID remains a challenge, our findings underscore the potential of biomarker-driven approaches for diagnosis and patient stratification. These insights are a crucial step towards developing targeted therapies to mitigate the long-term burden of post-COVID syndromes.

## Supporting information

S1 Fig Study flowchart showing the 3 study groups (PC, LC, pmC) at the 3 study visits (T0, T1, T2) and the parameters which were taken at each timepoint. (TIF)

S2 Fig Adapted TRIPOD-AI Checklist. The original TRIPOD-AI (Transparent Reporting of a multivariable prediction model for Individual Prognosis or Diagnosis – Artificial Intelligence) statement for clinical prediction models developed using artificial intelligence has been modified to align with the specific context and reporting requirements of this study.
(TIF)

S3 Fig Adapted STROBE Checklist. The original STROBE (Strengthening the Reporting of Observational Studies in Epidemiology) statement for observational studies.
(TIF)

S4 Fig Box plots of lung function parameters at T0 comparing LC and PC participants. At timepoint T0, lung function examination and routine laboratory analyses were performed in LC and PC participants. Results are depicted as boxplots showing the median as well as the first (Q1) and third quartiles (Q3), including p-values for group comparisons. **(A)** Fractionated exhaled nitric oxide (FeNO) measured in ppb. Normal value <20ppb **(B)** Forced expiratory volume in 1 second (FEV1) measured by body plethysmography. Normal value >80% of population-specific target value (measured by age, sex, height and weight). Statistical test: Mann-Whitney U test.
(TIF)

S5 Fig Box plots of standard laboratory parameters at T0 comparing LC and PC participants. At the timepoint T0 routine lab was performed in LC and PC participants and depicted in box plots showing the median as well as the first (Q1) and third quartiles (Q3), including p-values for group comparisons. **(A)** C-reactive protein (CRP) in the unit mg/L. Normal value <9,0 mg/L **(B)** Immunoglobulin E (IgE) in the unit kU/L. Normal value < 100kU/L **(C)** Lymphocytes in the unit G/l (10^9^/L). Normal value 1,4–4,8 x10^9^/ L **(D)** Eosinophils in the unit G/l (10^9^/L). Normal value 0,03–0,47 x10^9^/ L **(E)** Basophils in the unit G/l (10^9^/L). Normal value 0,01–0,07 x10^9^/ L **(F)** B-type natriuretic peptide (NT-proBNP) in the unit ng/L. Normal value <200ng/L. Statistical test: Mann-Whitney U test.
(TIF)

S6 Fig Volcano plots of upregulated OLINK proteins in different patient groups. Only parameters depicted in red are significant **(A)** Volcano plot of upregulated OLINK proteins in LC patients with and without the symptom fatigue **(B)** Volcano plot of upregulated OLINK proteins in LC patients with and without the symptom brainfog **(C)** Volcano plot of upregulated OLINK proteins in LC and PC patients with a higher and lower BMI of 30 kg/m^2^.
(TIF)

S7 Fig Associations between lung function parameters, routine laboratory and OLINK proteins. A matrix comparing various routine laboratory and lung function parameters, as well as selected OLINK proteins, was generated to visualize the relationships among these variables. Pearson’s method was applied to calculate pairwise correlations. Corresponding *p*-values are indicated (shown as asterixis with the gradation * < 0,05; ** < 0,01; *** < 0,001).
(TIF)

## Abbreviations

COVID-19: Coronavirus disease 2019
SARS-CoV2: Severe acute respiratory syndrome coronavirus type 2
PC: Post-severe COVID
LC: Long COVID
pmC: Post-mild COVID
T0: Timepoint beginning of rehabilitation
T1: Timepoint at two weeks after beginning of rehabilitation
T2: Timepoint at the end of rehabilitation
ICU: Intensive care unit
COPD: Chronic obstructive pulmonary disease
NA: Not available
DLCO: Diffusing capacity of the lung
FeNO: Exhaled nitric oxide
VC: Vital capacity of the lungs
FEV1: Forced expiratory volume
ECG: Electrocardiogram
6MWT: 6-minute walking test
CRP: C-reactive protein
NT-proBNP: N-terminal pro-B-type natriuretic peptide
IgE: Immunoglobulin E
LDH: Lactate dehydrogenase
IL6: Interleukin 6
OLINK: Olink Proteomics – Protein screening for biomarker discovery
PCR: Polymerase chain reaction
NPX: Normalized Protein Expression
PCA: Principal component analysis
BDCA2: Blood dendritic cell antigen 2
CD: Cluster of Differentiation
CKAP4: Cytoskeleton associated protein 4
CLEC4C/CLEC4A: C-type lectin domain family 4 member A and C
CSF-1: Colony stimulating factor 1
CXCL: Chemokine ligand
IFN: Interferon
IL: Interleukin
ITGA11: Integrin alpha-11
ITGB6: Integrin beta-6
JUN: Transcription factor AP-1
KRT19: Keratin 19
LAMP3: Lysosome-associated membrane glycoprotein 3
MCP-1: Monocyte Chemoattractant Protein 1
MMP-1: Matrix metalloproteinase 1
PD-L1: Programmed cell death ligand 1
PLXNA4: Plexin A4.
