# Supplementary figures and images for: Distinguishing post-COVID from long-COVID in adults: Development and validation of a biomarker signature using targeted proteomics and machine learning in a cross-sectional observational study
Source: PLoS One. 2026 Feb 27;21(2):e0338451. doi: 10.1371/journal.pone.0338451 (PMC12948100; doi:10.1371/journal.pone.0338451)

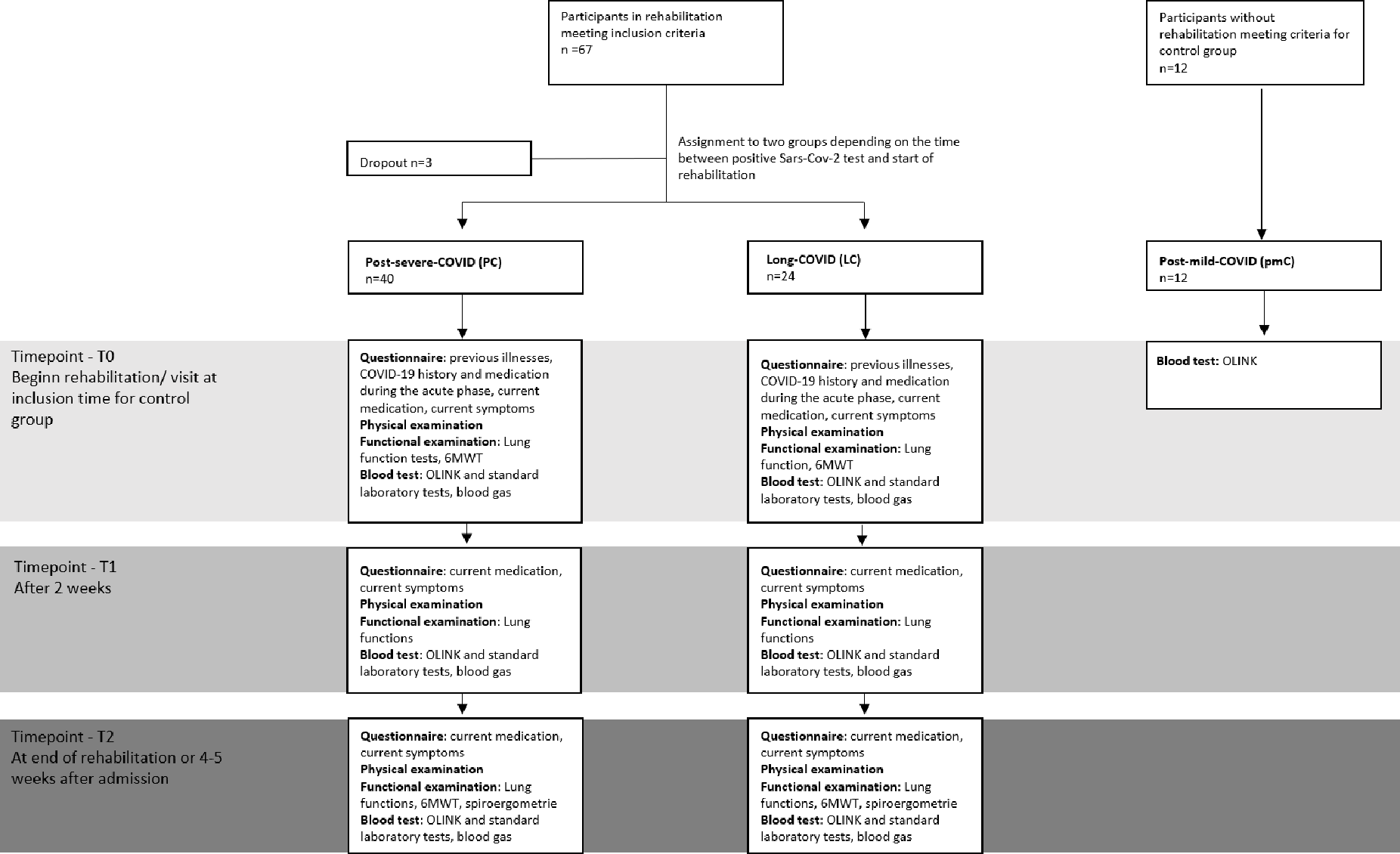

Supplement: S1 Fig — (TIF) [file pone.0338451.s001.tif]

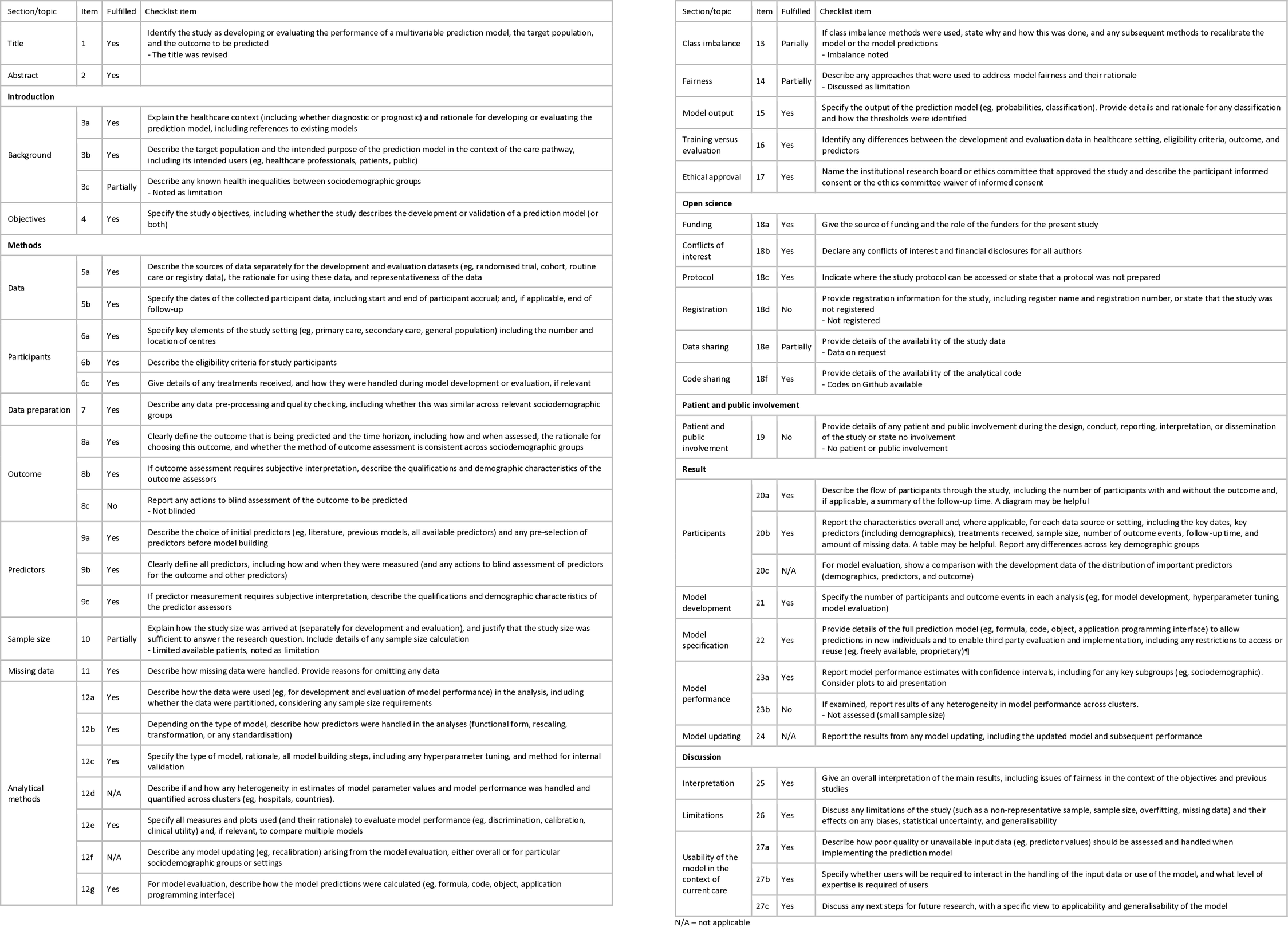

Supplement: S2 Fig — The original TRIPOD-AI (Transparent Reporting of a multivariable prediction model for Individual Prognosis or Diagnosis – Artificial Intelligence) statement for clinical prediction models developed using artificial intelligence has been modified to align with the specific context and reporting requirements of this study. (TIF) [file pone.0338451.s002.tif]

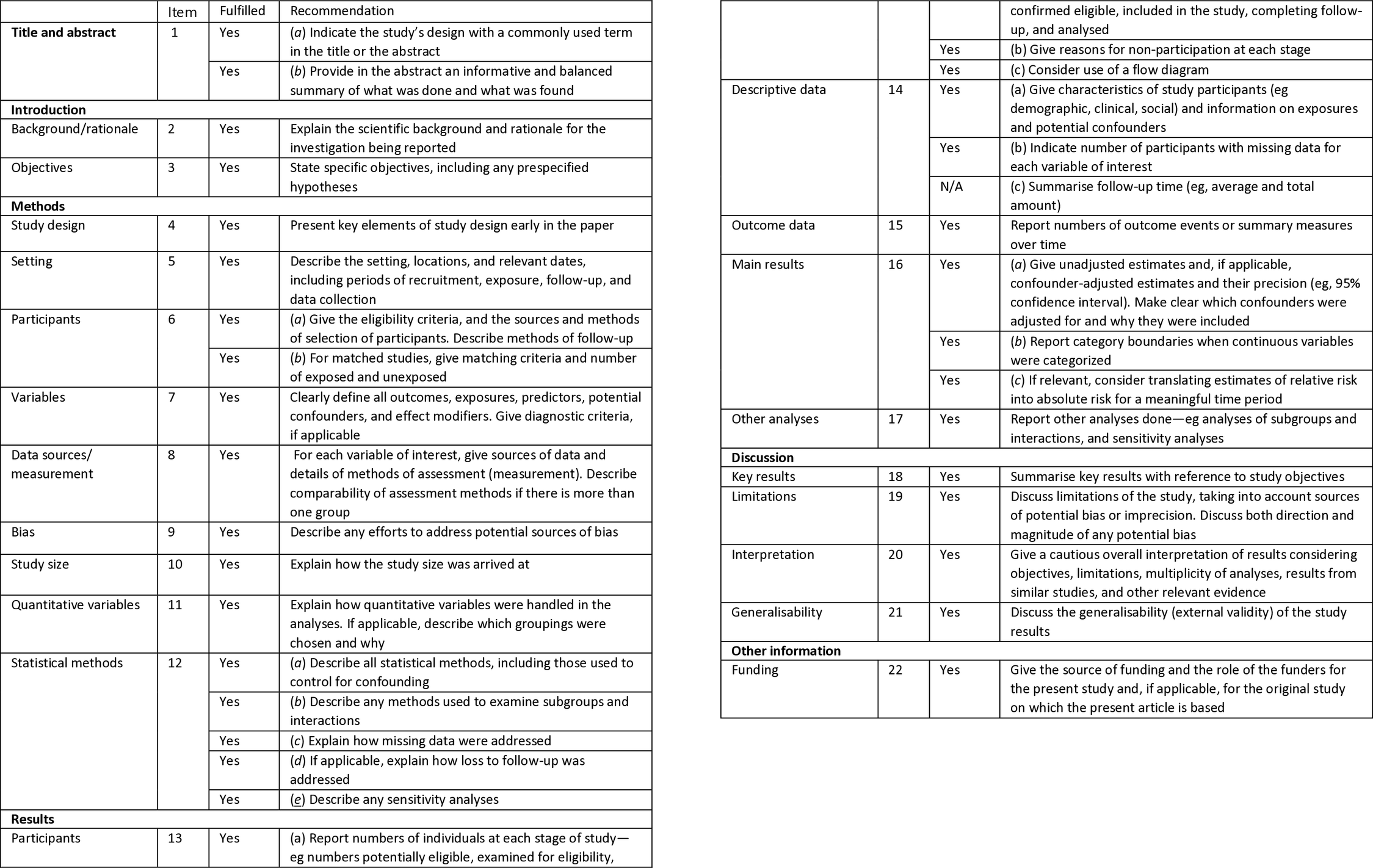

Supplement: S3 Fig — The original STROBE (Strengthening the Reporting of Observational Studies in Epidemiology) statement for observational studies. (TIF) [file pone.0338451.s003.tif]

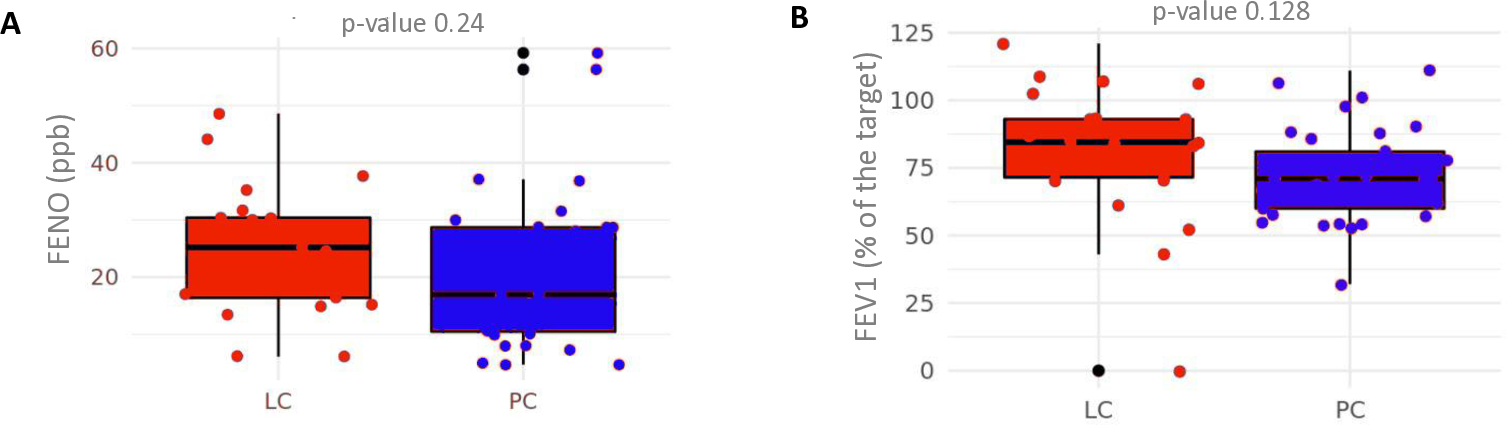

Supplement: S4 Fig — At timepoint T0, lung function examination and routine laboratory analyses were performed in LC and PC participants. Results are depicted as boxplots showing the median as well as the first (Q1) and third quartiles (Q3), including p-values for group comparisons. (A) Fractionated exhaled nitric oxide (FeNO) measured in ppb. Normal value <20ppb (B) Forced expiratory volume in 1 second (FEV1) measured by body plethysmography. Normal value >80% of population-specific target value (measured by age, sex, height and weight). Statistical test: Mann-Whitney U test. (TIF) [file pone.0338451.s004.tif]

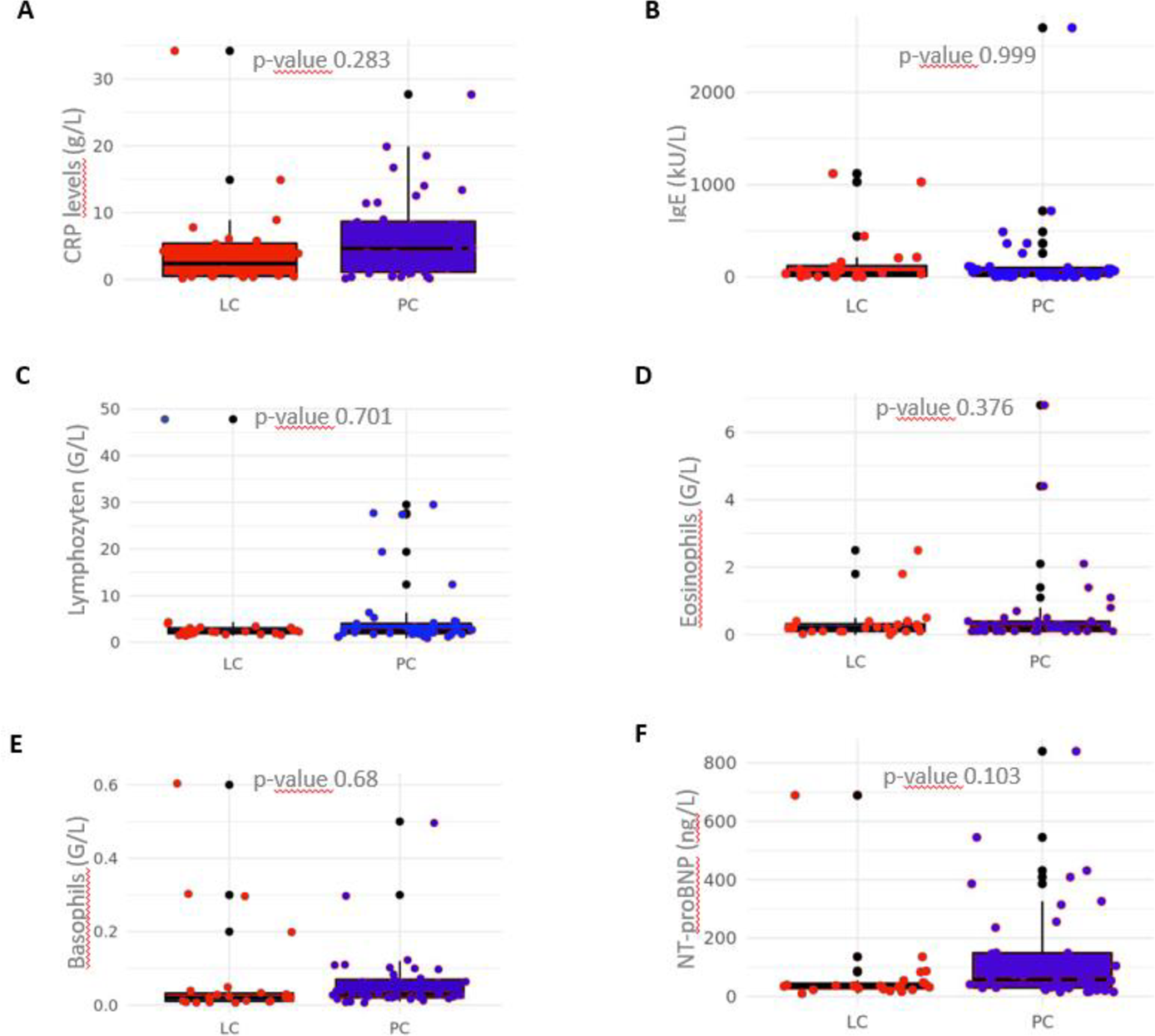

Supplement: S5 Fig — At the timepoint T0 routine lab was performed in LC and PC participants and depicted in box plots showing the median as well as the first (Q1) and third quartiles (Q3), including p-values for group comparisons. (A) C-reactive protein (CRP) in the unit mg/L. Normal value <9,0 mg/L (B) Immunoglobulin E (IgE) in the unit kU/L. Normal value < 100kU/L (C) Lymphocytes in the unit G/l (109/L). Normal value 1,4–4,8 x10^9^/ L (D) Eosinophils in the unit G/l (109/L). Normal value 0,03–0,47 x10^9^/ L (E) Basophils in the unit G/l (109/L). Normal value 0,01–0,07 x10^9^/ L (F) B-type natriuretic peptide (NT-proBNP) in the unit ng/L. Normal value <200ng/L. Statistical test: Mann-Whitney U test. (TIF) [file pone.0338451.s005.tif]

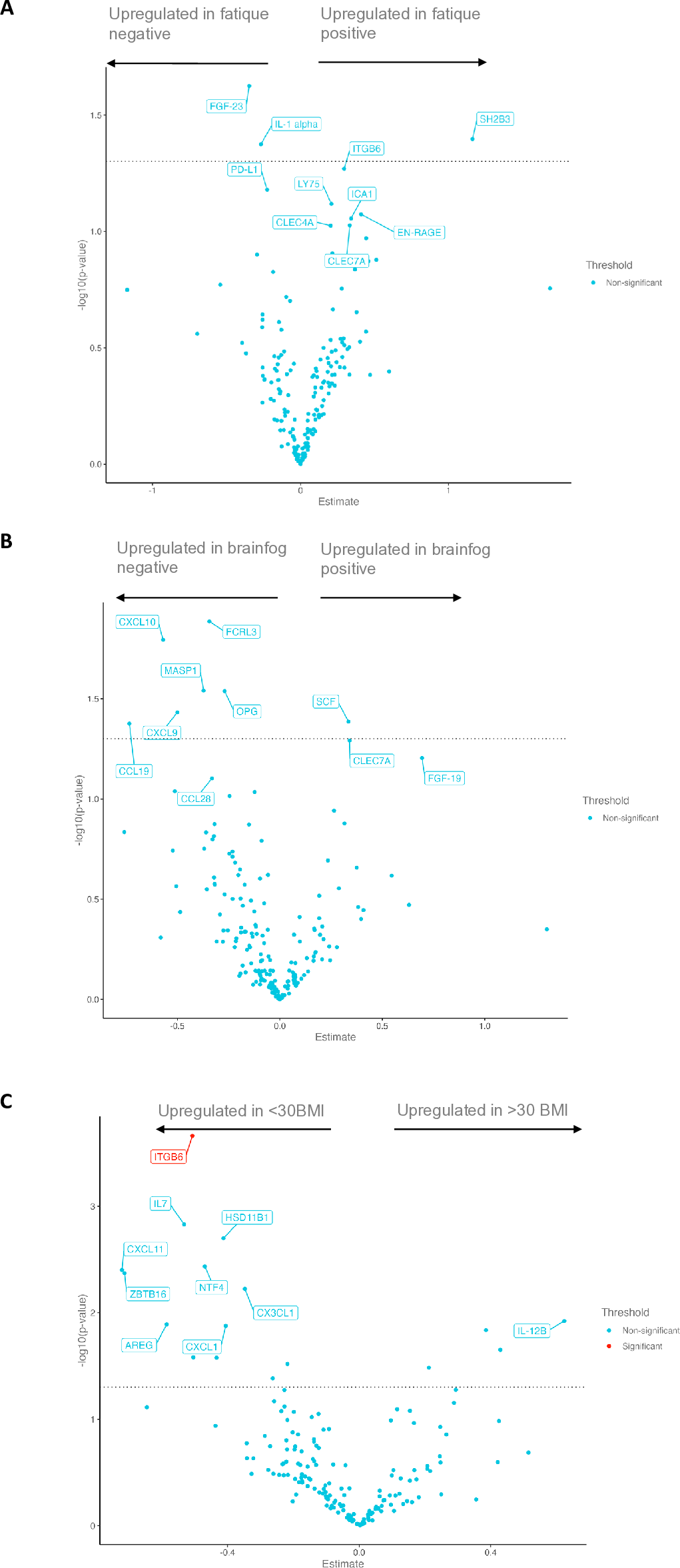

Supplement: S6 Fig — Only parameters depicted in red are significant (A) Volcano plot of upregulated OLINK proteins in LC patients with and without the symptom fatigue (B) Volcano plot of upregulated OLINK proteins in LC patients with and without the symptom brainfog (C) Volcano plot of upregulated OLINK proteins in LC and PC patients with a higher and lower BMI of 30 kg/m2. (TIF) [file pone.0338451.s006.tif]

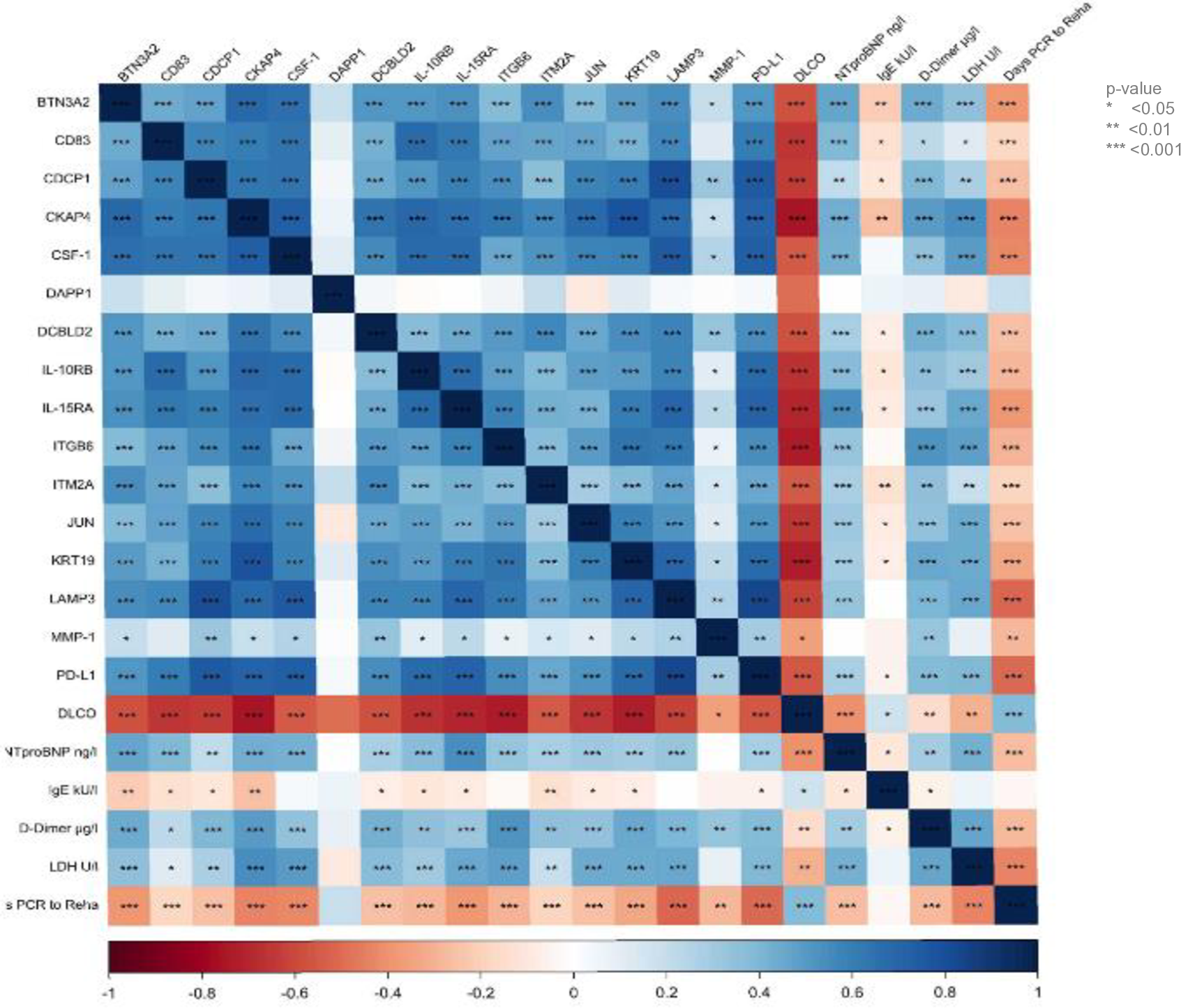

Supplement: S7 Fig — A matrix comparing various routine laboratory and lung function parameters, as well as selected OLINK proteins, was generated to visualize the relationships among these variables. Pearson’s method was applied to calculate pairwise correlations. Corresponding p-values are indicated (shown as asterixis with the gradation * < 0,05; ** < 0,01; *** < 0,001). (TIF) [file pone.0338451.s007.tif]
